# Improved identification of abdominal aortic aneurysm using the Kernelized Expectation Maximization algorithm

**DOI:** 10.1098/rsta.2020.0201

**Published:** 2021-06-28

**Authors:** Daniel Deidda, Mercy I. Akerele, Robert G. Aykroyd, Marc R. Dweck, Kelley Ferreira, Rachael O. Forsythe, Warda Heetun, David E. Newby, Maaz Syed, Charalampos Tsoumpas

**Affiliations:** ^1^ National Physical Laboratory, Teddington, UK; ^2^ Biomedical Imaging Science Department, University of Leeds, Leeds, UK; ^3^ Department of Statistics, University of Leeds, Leeds, UK; ^4^ Edinburgh Imaging Facility, Queen’s Medical Research Institute, Edinburgh, UK; ^5^ British Heart Foundation Centre for Cardiovascular Science, University of Edinburgh, Edinburgh, UK; ^6^ Department of Radiology, Weill Cornell Medicine, New York, NY, USA

**Keywords:** PET/CT, kernel method, PET, aortic aneurysm

## Abstract

Abdominal aortic aneurysm (AAA) monitoring and risk of rupture is currently assumed to be correlated with the aneurysm diameter. Aneurysm growth, however, has been demonstrated to be unpredictable. Using PET to measure uptake of [^18^F]-NaF in calcified lesions of the abdominal aorta has been shown to be useful for identifying AAA and to predict its growth. The PET low spatial resolution, however, can affect the accuracy of the diagnosis. Advanced edge-preserving reconstruction algorithms can overcome this issue. The kernel method has been demonstrated to provide noise suppression while retaining emission and edge information. Nevertheless, these findings were obtained using simulations, phantoms and a limited amount of patient data. In this study, the authors aim to investigate the usefulness of the anatomically guided kernelized expectation maximization (KEM) and the hybrid KEM (HKEM) methods and to judge the statistical significance of the related improvements. Sixty-one datasets of patients with AAA and 11 from control patients were reconstructed with ordered subsets expectation maximization (OSEM), HKEM and KEM and the analysis was carried out using the target-to-blood-pool ratio, and a series of statistical tests. The results show that all algorithms have similar diagnostic power, but HKEM and KEM can significantly recover uptake of lesions and improve the accuracy of the diagnosis by up to 22% compared to OSEM. The same improvements are likely to be obtained in clinical applications based on the quantification of small lesions, like for example cancer.

This article is part of the theme issue ‘Synergistic tomographic image reconstruction: part 1’.

## 1. Introduction

Abdominal aortic aneurysm (AAA) monitoring and rupture prediction is currently based on the measurement of the aneurysm diameter over time to determine its growth [1]. Aneurysm growth, however, has been demonstrated to be difficult to predict and affected by many biological factors [2]. To overcome this issue, the use of molecular imaging such as positron emission tomography (PET)/computed tomography (CT) with [^18^F]-sodium fluoride (NaF) as the radiotracer was proposed and investigated [3]. Measured uptake of [^18^F]-NaF in calcified lesions of the abdominal aorta has been demonstrated to be useful to identify AAA and to predict its growth [4].

These results were obtained using the standard ordered subsets expectation maximization (OSEM) [5] as image reconstruction method, including point spread function (PSF) modelling, and post-reconstruction filtering. As mentioned above, PET allows identification of micro-calcification in the aorta, but low spatial resolution and the partial volume effect (PVE) pose a challenge to this task. In addition, [^18^F]-NaF shows high activity in the spine which is extremely close to the aneurysm and can cause spill-in from the spine to the aorta [6]. PVE can lead to overestimation (spill-in) or underestimation (spill-out) of activity in small regions, especially when the target region is close to a hot background region. The gold standard OSEM with PSF modelling has been shown to suffer from this problem and algorithms like the background correction and the kernel method can reduce the spill-in effect [7].

The kernel method for image reconstruction has been demonstrated to improve noise suppression while preserving activity of small lesions, to be robust with respect to anatomical-functional inconsistencies, and to improve quantification in different case scenarios [8–11]. Based on these results, the assumption of this study is that the early diagnosis of the AAA could be improved by using reconstruction techniques that preserve small activity lesions such as the Hybrid Kernelized EM (HKEM).

The paper is structured as follows: §2. describes the datasets used in this study, the image reconstruction settings, and the experimental methodology. Section 3. presents the results of the proposed method and comparison of results obtained from the different algorithms. The results are discussed in §4. and conclusion is drawn in §5.

## 2. Methods and materials

### (a) Kernel description

Following the mathematical notation in [9], the algorithm is described as follows.

Considering the kernel method for machine learning [12], we can describe the PET image, *λ*, as a linear combination

2.1 $$\lambda_{j}=\sum_{f=1}^{N_{j}}\alpha_{f}k_{fj};$$

where *k*_*fj*_ is the *fj*th element of the kernel matrix, *k*, *α*_*f*_ is the *f*th element of the coefficient vector that we need to estimate, and *N*_*j*_ is the number of feature vectors used to estimate the kernel element relative to the image voxel *j*. After the maximization of the log-likelihood for *α* we obtain the following iterative algorithm

2.2 $$\alpha_{f}^{\left(n+1\right)}=\frac{\alpha_{f}^{\left(n\right)}}{\sum_{j=1}^{N_{f}}k_{fj}^{\left(n\right)}\sum_{i\in J_{f}}c_{ij}}\sum_{j=1}^{N_{f}}k_{fj}^{\left(n\right)}\sum_{i=1}^{L}c_{ij}\frac{y_{i}}{\sum_{l\in I_{i}}c_{il}\sum_{q=1}^{N_{l}}k_{ql}^{\left(n\right)}\alpha_{q}^{\left(n\right)}+s_{i}};$$

where $\alpha_{f}^{\left(n\right)}$ is the *f*th kernel coefficient estimated at iteration *n*, *y*_*i*_ is the *i*th line of response (LOR), *L* is the total number of LORs, *c*_*ij*_ is the probability that an event occurring in voxel *j* produces a coincidence in the *i*th LOR and *s*_*i*_ is the additive term introducing the randoms and scatter correction for the *i*th LOR.

The kernel matrix, *K*^(*n*)^ is the product of two different terms, *K*_*m*_ and $K_{p}^{\left(n\right)}$, and allows modelling of prior information from the anatomical and functional images respectively

2.3 $$K^{\left(n\right)}=K_{m}\cdot K_{p}^{\left(n\right)};$$

with

2.4 $$k_{m}(v_{j},v_{f})=\exp\left(-\frac{||v_{j}-v_{f}|{|}^{2}}{2\sigma_{m}^{2}}\right)\exp\left(-\frac{||x_{j}-x_{f}|{|}^{2}}{2\sigma_{dm}^{2}}\right)$$

and

2.5 $$k_{p}(z_{j}^{\left(n\right)},z_{f}^{\left(n\right)})=\exp\left(-\frac{||z_{j}^{\left(n\right)}-z_{f}^{\left(n\right)}|{|}^{2}}{2\sigma_{p}^{2}}\right)\exp\left(-\frac{||x_{j}-x_{f}|{|}^{2}}{2\sigma_{dp}^{2}}\right)$$

**Figure 1.**
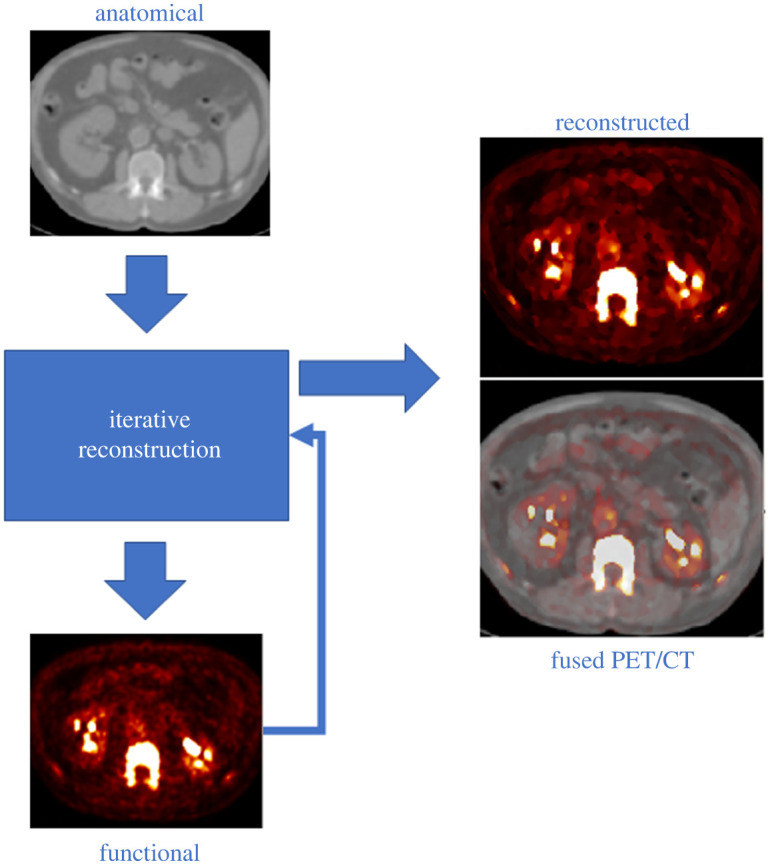
Schematic of the reconstruction with the HKEM. The anatomical image is used as prior information in the reconstruction algorithm; the result of this iteration is used as extra prior information for the following iteration. (Online version in colour.)

***x***_*j*_ is the coordinate of the *j*th voxel, ***v***_*j*_ and $z_{j}^{\left(n\right)}$ are the feature vectors calculated, respectively, from the anatomical image and the *n*th update image, *α*^*n*^, while *σ*_*m*_, *σ*_*p*_, *σ*_*dm*_ and *σ*_*dp*_ are scaling parameters for the distances in (2.4) and (2.5), which allow adjustment of noise suppression and edge preservation. The anatomical information in this work is the CT image, which shows the spine and the aorta at high resolution. The reconstruction process is schematically described in figure 1.

KEM can be thought of as a special condition of HKEM when no iterative information is used. The objectives of this work are to investigate the usefulness of the KEM and HKEM in improving the diagnosis of AAA with a sample of 61 positive patients and 11 control patients; to compare HKEM, KEM and OSEM; and to investigate the optimization of the kernel parameter settings. The latter was achieved using a sub-sample of 10 patients. The sub-sample was randomly selected from the positive patient datasets and was excluded from the test dataset for the analysis.

### (b) Patient data

A sample of 61 PET/CT datasets from patients showing aneurysm of varying diameters, and 11 PET/CT control datasets were used from the archive of the [18F]-NaF uptake in AAA (SoFIA3) PET/CT study (NCT02229006) [4]. The study considered patients with an average age of 72.5±6.9 years, body mass index 27.6 ± 3.5 kg m^−2^ and aortic diameter 48.8 ± 7.7 mm.

Each patient was injected with 125 MBq of [^18^F]-NaF and imaged 60 min post-injection using the Biograph mCT scanner (Siemens Healthineers, Knoxville, TN, USA) [3]. A low-dose CT attenuation correction (CTAC) acquisition was carried out (120 kV, 50 mAs, 5/3 mm) followed by acquisition of PET data using 3 × 10 min bed positions to ensure coverage from the thoracic aorta to the aortic bifurcation.

### (c) Reconstruction

All datasets were reconstructed with 21 subsets and 10 full iterations using HKEM, KEM and OSEM. The most frequent optimum parameter settings (*N*, *σ*_*m*_, *σ*_*p*_, *σ*_*dm*_ and *σ*_*dp*_) for KEM and HKEM were evaluated on a sub-sample of 10 positive patients. For all the algorithms point spread function (PSF) modelling was incorporated as an isotropic three-dimensional Gaussian kernel with 4.4 mm full width half maximum (FWHM) for all directions. A Gaussian post-reconstruction filter with 5 mm FWHM was applied to OSEM. For this reason we refer to it as OSEM+G in all the figures.

The iteration number used for the comparison and the images in the figures was chosen as the one providing the highest number of true positives. The reconstructed PET image size was 400×400×109 voxels, and the voxel size was 2.04 × 2.04 × 2.03 cm^3^. The CT image was used as anatomical information for KEM and HKEM, and it was down-sampled to match the PET images.

Scatter, randoms, normalization and attenuation corrections were estimated using the vendor software. The image reconstruction was made using the open source Software for Tomographic Image Reconstruction (STIR) [13] v. 4.0.

### (d) Image analysis

**Figure 2.**
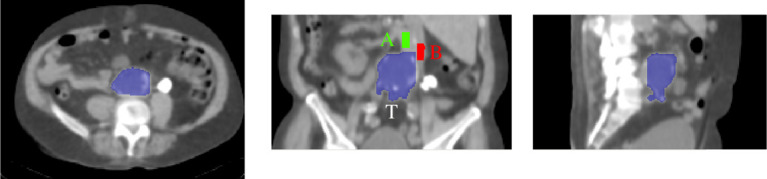
Extracted regions of interest (ROIs) showed on the CT image: the target AAA region (T), non-AAA aorta (A) and blood pool region or vena cava (B). (Online version in colour.)

A database containing all the quantitative information for each patient and region of interest (ROI) was created using STIR [14], and all the statistical analysis was performed with R [15]. The comparison was carried out in terms of different metrics, following the clinical protocol in [4]. ROI analysis was performed using three separate regions: (1) the target (T) AAA ROI where the micro-calcification were expected to be, (2) the part of the aorta that does not show growth nor activity (A), and (3) the vena cava (B). An example of the segmented ROIs is shown in figure 2. For the control group, even though the aorta should not have enlargement, the part of the artery where the aneurysm is usually located was considered for the ROI T. This is the part close to the aorta bifurcation.

The ROIs were extracted using the CT image, at the same time it was possible to display the PET image. In this way, it was possible to exclude those voxels that were too close to the spine and therefore to avoid spill-in effects in the analysis. A semi-automatic segmentation method in ITK-SNAP based on thresholding [16] was used. Quantitative comparison was performed using the maximum target to blood pool ratio (TBR_max_) [17]:

2.6 $${\text{TBR}}_{\text{max}}(T)=\frac{{\text{SUV}}_{\text{max}}(T)}{{\text{SUV}}_{\text{mean}}(B)};$$

where SUV_max_(*T*) is the maximum SUV value within T, SUV_mean_(*B*) is the mean value within the blood pool region, B. The TBR_max_(*A*) is calculated similarly. Then the percentage increase of uptake between the aneurysm and the normal part of the aorta was calculated as

2.7 $$\text{Increase}=\frac{{\text{TBR}}_{\text{max}}(T)-{\text{TBR}}_{\text{max}}(A)}{{\text{TBR}}_{\text{max}}(A)}\times 100.$$

An increase higher than 25% is considered significant according to the European Organization for Research and Treatment of Cancer (EORTC) [18–22]. If the exam shows such an increase the patient is considered positive for AAA. To assess the significance of the difference between the quantification with the three algorithms a paired *t*-test was performed for each algorithm combination. A receiver operating characteristic (ROC) analysis was also performed to compare the accuracy of the techniques at the standard 25% threshold using the open source pROC package [23], including the Delong’s test to assess the differences between the area under the curve (AUC). Pearson’s correlation analysis was carried out to assess the relationship between the estimated uptake increase and the diameter of the aneurysm. Finally, a predictive model was created using logistic regression. To perform this analysis, random sub-sampling was used to make sure that each class has around the same number of cases. The regression studies the relationship between ROI value (TBRmax) and the AAA positivity.

## 3. Results

**Figure 3.**
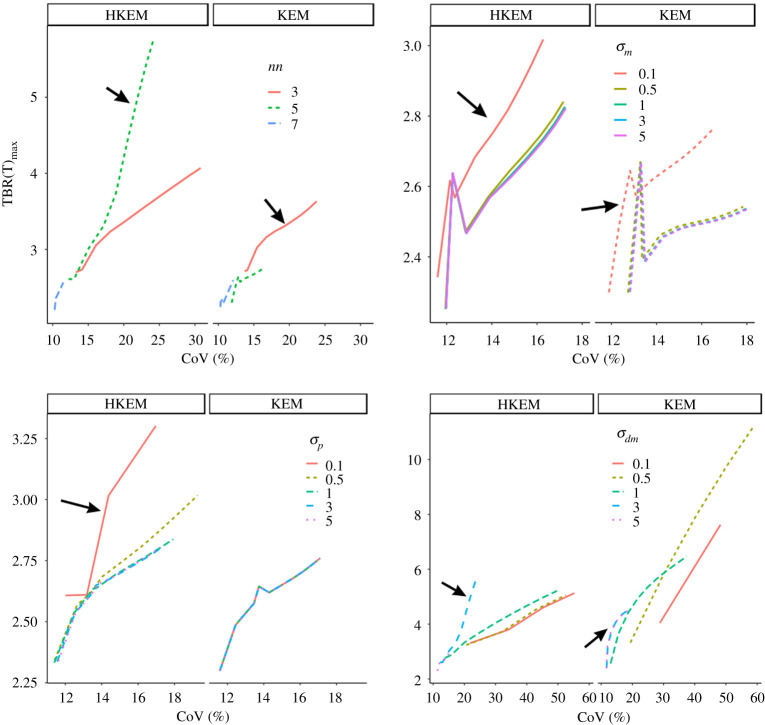
Example of the parameter optimization process using the results for one patient. (Online version in colour.)

To find the optimum parameter setting for HKEM and KEM, a sub-sample of 10 patients was used. These data were reconstructed with a set of different parameters combination and TBR_max_(*T*) was plotted against the CoV as reported in figure 3. Figure 3 illustrates an example of how the optimum kernel setting was selected for each of the 10 patients. The optimum parameter value was the one that gave higher TBR_max_ at fixed coefficient of variation (CoV). This procedure was repeated for all the 10 patient datasets selected for the optimization study and table 1 reports the frequency of the optimum kernel parameter value for the selected data using KEM and HKEM. The table highlights in bold the highest frequency. Note that *σ*_*p*_ is specific to HKEM as it controls the edge preservation of the functional information.

**Table 1.** Frequencies of optimum value among 10 patients for each kernel parameter. *σ*_*p*_ is specific to HKEM as it controls the edge preservation of the functional information.

*nn*	HKEM	KEM	*σ*_*m*_	HKEM	KEM	*σ*_*p*_	HKEM	*σ*_*dm*_	HKEM	KEM
3	3	**6**	0.1	**10**	**7**	0.1	**6**	0.1	2	2
5	**7**	4	0.5		1	0.5	3	0.5	0	1
7			1			1	1	1	2	3
			3			3		3	**6**	**4**
			5		2	5		5		

**Figure 4.**
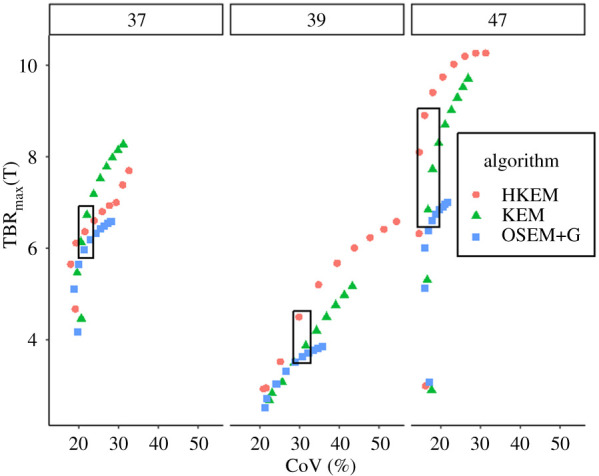
TBR_max_(*T*) as a function of the CoV (and iteration) for all the algorithms using three patients. The numbers on the top are identification numbers assigned for this study, and the box shows the points where the different algorithms have comparable CoV. (Online version in colour.)

Figure 4 shows TBR_max_(*T*) as a function of the CoV and the iteration number. The black box highlights the iteration which gave similar CoV among the three algorithms.

**Figure 5.**
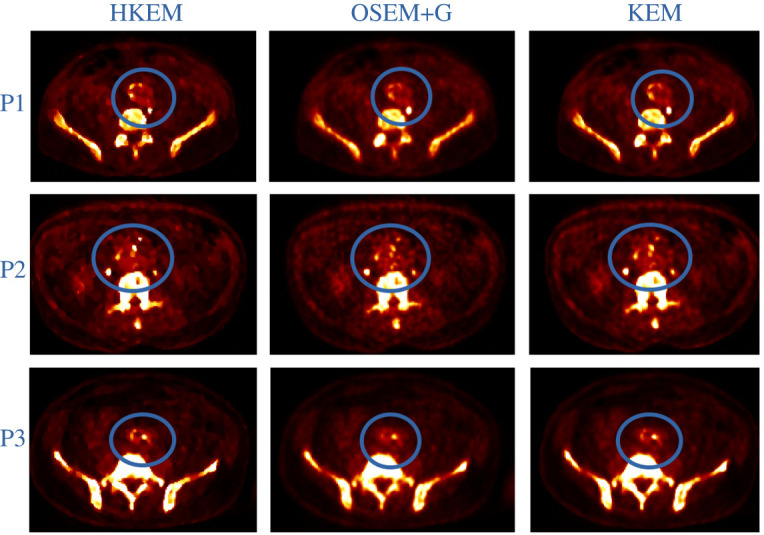
Comparison of the images reconstructed with HKEM, OSEM+G and KEM for three patients. The images show a transverse view through the abdomen, highlighting the AAA region within the circle. (Online version in colour.)

Figure 5 reports the reconstructed images for three patient datasets and compares between the different algorithms. The circles highlight the aneurysm with the calcified lesions.

**Figure 6.**
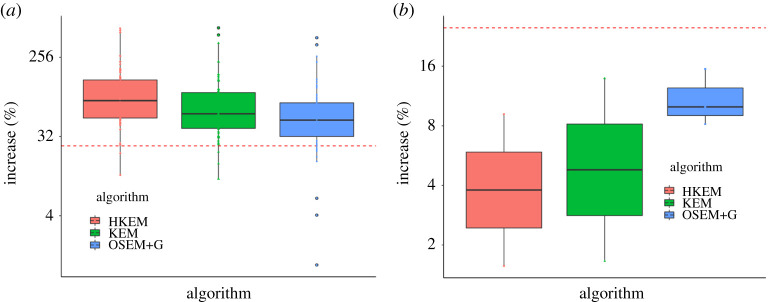
Increase of uptake between the ROI T and A: (*a*) for the AAA patient data, (*b*) for the control group. The dashed line represents the 25% increase that defines AAA positivity. (Online version in colour.)

The analysis on the full sample of 61 AAA patients and 11 control group is reported in figure 6. In particular, (a) reports the percentage increase in TBR_max_ for all techniques and AAA patients, whereas (b) reports the same for the control group. The red dashed line is the 25% threshold used to classify the patient as positive to AAA or not.

Table 2 reports the results of the paired *t*-test showing the significance of the differences between HKEM and OSEM+G, KEM and OSEM+G, and HKEM and KEM at the confidence level (CL) of 95%.

**Table 2.** Paired *t*-test assessing the difference between the results obtained with the three algorithms (95% CL).

algorithms	*p*-value
HKEM - OSEM+G	7 · 10^−6^
KEM - OSEM+G	1.8 · 10^−4^
HKEM - KEM	8.4 · 10^−4^

An ROC curve analysis was carried out to assess the diagnostic power of the three methods. Table 3 reports the values of specificity, sensitivity, accuracy and precision for the threshold value of 25%, and the area under the curve for HKEM, KEM and OSEM+G. The ROC curves are reported in the electronic supplementary material, figure S1.

**Table 3.** ROC analysis and comparison between HKEM, KEM, OSEM+G.

algorithm	specificity	sensitivity	accuracy	precision	AUC
HKEM	1	0.96	0.97	1	1
KEM	1	0.94	0.95	1	0.998
OSEM+G	1	0.77	0.81	1	0.972

**Figure 7.**
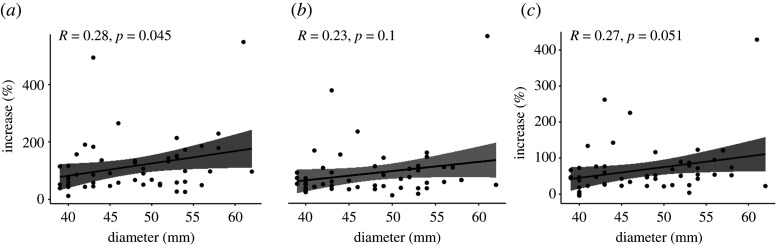
Correlation analysis for (*a*) HKEM, (*b*) KEM, (*c*) OSEM+G (95% CL).

Figure 7 shows the results of Pearson’s correlation analysis to study the relationship between the uptake increase and the diameter of the aneurysm.

**Figure 8.**
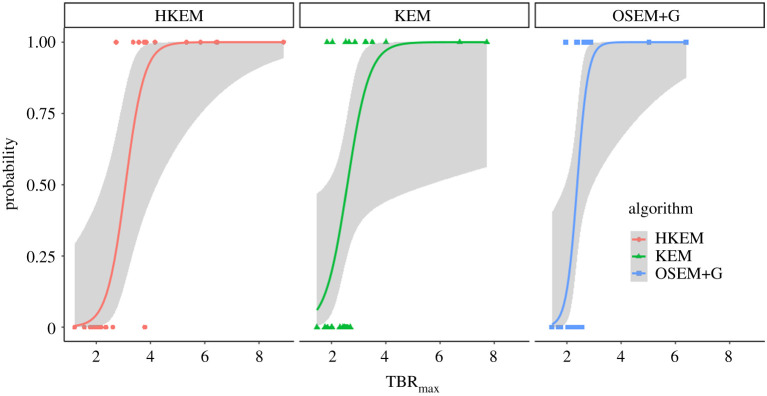
Logistic regression fit with standard error for each algorithm using the balanced data. The scattered points represent the true value of positivity (1 for positive or 0 negative) against the TBR_max_. (Online version in colour.)

Finally, figure 8 and table 4 report the logistic regression analysis, included coefficients, residual deviances, standard errors and *p*-values for each algorithm.

**Table 4.** Logistic regression analysis and comparison between HKEM, KEM, OSEM+G.

algorithm	intercept/*p*-value	TBR_max_ coeff/*p*-value	accuracy	residual deviance
HKEM	−8.86 ± 3.6/0.02	2.8 ± 1.2/0.02	0.91	10.0
KEM	−6.4 ± 3/0.04	2.4 ± 1.2/0.04	0.77	20.6
OSEM+G	−11.9 ± 5.4/0.03	5.0 ± 2.3/0.03	0.77	18.96

## 4. Discussion

PET imaging with [^18^F]-NaF is presently being investigated for the classification of micro-calcification in arteries. The low spatial resolution and the PVE, however, threaten the diagnostic accuracy in cases where the calcification is small, making it challenging to detect the disease at early stage. This work investigates the benefits of the recently proposed algorithms, KEM and HKEM, on the quantification and classification of AAA.

The choice of the iteration is a challenging task. As can be seen from figure 4 the algorithms can have different CoV at the same iteration so it would be fair to compare the algorithms at the same noise level. The analysis was first carried out by selecting the iteration in a way that all methods were compared at similar CoV for each patient. Nevertheless, such a procedure would need to be repeated for every patient and in a clinical environment it would not be possible to look at the different performance for each iteration since the vendor software only saves the chosen iteration image. For this reason, all the information was saved for 10 iterations and the iteration with the highest number of true positives was selected for every algorithm. The selected iteration was the 4th for HKEM and KEM and the 3rd for OSEM+G. The outcomes of these two different choices of iteration were the same. From the same figure, it can be noted that the TBR_max_ value keeps increasing iteration after iteration and the algorithms do not reach a plateau within 10 full iterations. This is consistent with all the patients, and is due to the fact that the analysis is based on the voxel with the maximum value.

The visual comparison in figure 5, for three patients, shows how HKEM is able to provide well-defined lesions in the aneurysm compared to the other two algorithms. KEM also shows improved definition and higher contrast over OSEM+G. Although only three patient images are reported, these results were consistent among all the patient datasets. These results give confirmation of what was previously demonstrated in other work [9,24] but on a larger scale.

Almost all the positive patients in the test dataset, 96% for HKEM, 94% for KEM and 76% for OSEM+G, showed significant (relative increase >25%) uptake in the aneurysm. Figure 6 shows that on average KEM and HKEM provide a higher increase than OSEM+G and the statistical significance of these differences was demonstrated with a paired *t*-test at the 95% CL. From table 2, it is evident that the TBR_max_(*T*) estimated with one algorithm is different to the one obtained with the other two algorithms, with a *p*-value lower than 0.01.

The ROC analysis is reported in table 3 for the threshold value of 25%. The specificity value tells us that all algorithms provide the maximum probability of identifying a non-diseased patient as negative. The precision is the same for all the algorithms. The sensitivity, which describes the probability of a diseased patient being identified as positive, provides the real difference between the algorithms with the highest value for HKEM followed by KEM with a difference of 2%, and OSEM+G, with a difference of 18%. The accuracy also shows a similar trend, the HKEM and KEM provide respectively a 16% and 14% higher probability of classifying a patient with the correct label relative to OSEM+G. It can be seen that the value of the AUC is similar among the algorithms and is always close to 1. Delong’s test, used to compare whether each pair of AUC are different, provided a *p*-value higher than 0.06, meaning that although the algorithms behave differently for each threshold, their performances are in agreement globally (at the 95% CL).

The final test was the study of the correlation between the diameter of the aneurysm and the increase of uptake between the aneurysm and the normal aorta. Previous studies have reported non-significant correlation at the 95% CL [4,6] using OSEM+G, and this work is in agreement with these findings as reported in figure 7. In fact, the correlation is not significant for OSEM+G and KEM at the 95% CL. For HKEM, in contrast, the two variables are significantly correlated with a *p*-value of 0.045. The correlation is nevertheless moderate with a coefficient of 0.28. In addition, it is worth noting that the OSEM+G *p*-value is just at the limit and the correlation would be significant at the 90% CL. These results show the existence of a weak relationship between aneurysm size and uptake increase, but it is not enough to use the measurement of the aneurysm size as the only biomarker because small aneurysm may still have micro-calcification which is detectable using PET. The reader can notice that there are outliers in figure 6, although these CT and PET images did not show particular differences to those of other patients, to check robustness, the analysis was repeated excluding the outliers and the results were unchanged. Figure 8 and table 4 are useful to cross-validate the study. It can be seen that the ROI value is a significant parameter for each algorithm, with *p*-value ≤0.04, and that the HKEM achieves the highest accuracy and the smallest residual deviance. Based on the estimated probability as a function of TBR_max_ we can predict for each algorithm which value of TBR_max_ is the classification threshold. This value is 3.13 for HKEM, 2.69 for KEM and 2.26 for OSEM+G.

Even though this study was optimized and designed for the identification of calcified lesions in the AAA, its results show promising implications on all diagnostic applications where relatively small lesions need to be detected and quantified. Synergistic reconstruction algorithms like KEM and HKEM, with optimized hyper-parameters, not only have similar diagnostic performance to OSEM+G, without loss of information, but also significantly improve the quantification of the lesions and the accuracy of the diagnostic prediction. It is worth noting that HKEM relies on previous activity estimate, and although previous studies [9,25] have shown that it converges to a fixed point, no theoretical convergence guarantee can be provided.

If the sensitivity of PET scanners is substantially higher, as for example in the case of long axial field-of-view scanners [26], one could reconstruct the PET images matching the CT voxel size, and consequently gaining substantially more resolution improvement.

With the rising utilization of deep learning in image reconstruction, an extension of this work could be to train a network to find the optimum parameter setting, although a larger amount of data may be needed.

## 5. Conclusion

The performance of two synergistic reconstruction algorithms, HKEM and KEM with the clinical gold standard, OSEM+G, was compared on the issue of AAA identification and uptake quantification. Sixty-one AAA and 11 control datasets were involved in the study. The statistical analysis demonstrated that HKEM, which makes use of both anatomical and functional information, is able to provide generally higher uptake increase. KEM, which uses only anatomical information, still provided significant uptake increase compared to OSEM+G; all algorithms have excellent diagnostic power but show differences in sensitivity and accuracy at the recommended threshold of 25%, with HKEM and KEM providing the highest values. In the light of these results we are able to assert that synergistic reconstruction algorithms such as HKEM and KEM, do bring benefit to the diagnosis of a disease like AAA by reducing the rate of false positives and false negatives.

## Data accessibility

The comma separated value files generated for the analysis and containing the ROI mean, min, max, SD and CoV values for all patients, reconstruction algorithms, and ROIs are stored in the following repositories: 0.5281/zenodo.3974641.
